# Quality of Life Assessment Using the WHOQOL-BREF Survey in Hospitalized Patients with Alcohol Use Disorder from Romania

**DOI:** 10.3390/diseases12070158

**Published:** 2024-07-16

**Authors:** Melania Lavinia Bratu, Dorel Sandesc, Teodora Anghel, Liana Dehelean, Mariana Bondrescu, Felix Bratosin, Raluca Tudor

**Affiliations:** 1 Center for Neuropsychology and Behavioral Medicine, Department of Psychology, Faculty of General Medicine, “Victor Babes” University of Medicine and Pharmacy Timisoara, Eftimie Murgu Square 2, 300041 Timisoara, Romania; bratu.lavinia@umft.ro; 2 Doctoral School, “Victor Babes” University of Medicine and Pharmacy Timisoara, Eftimie Murgu Square 2, 300041 Timisoara, Romania; 3 Center for Cognitive Research in Neuropsychiatric Pathology, Department of Neurosciences, “Victor Babes” University of Medicine and Pharmacy Timisoara, Eftimie Murgu Square 2, 300041 Timisoara, Romania; lianadeh@umft.ro (L.D.); mariana.bondrescu@umft.ro (M.B.); 4 Department of Anesthesia and Intensive Care, “Victor Babes” University of Medicine and Pharmacy Timisoara, Eftimie Murgu Square 2, 300041 Timisoara, Romania; sandesc.dorel@umft.ro; 5 Department of Neurosciences-Psychiatry, “Victor Babes” University of Medicine and Pharmacy Timisoara, Eftimie Murgu Square 2, 300041 Timisoara, Romania; 6 Methodological and Infectious Diseases Research Center, Department of Infectious Diseases, “Victor Babes” University of Medicine and Pharmacy Timisoara, Eftimie Murgu Square 2, 300041 Timisoara, Romania; felix.bratosin@umft.ro; 7 Second Discipline of Neurology, “Victor Babes” University of Medicine and Pharmacy Timisoara, Eftimie Murgu Square 2, 300041 Timisoara, Romania; tudor.raluca@umft.ro

**Keywords:** alcohol addiction, alcohol use disorder, quality of life, psychiatry

## Abstract

This cross-sectional analysis aimed to assess the quality of life (QoL) among hospitalized patients with alcohol use disorder (AUD) in Romania, utilizing the WHOQOL survey. Conducted from January to December 2023 in the Psychiatry Clinic of the “Pius Brinzeu” Emergency Clinical Hospital in Timisoara, this study engaged 70 participants, adhering to ethical standards outlined in the Declaration of Helsinki. Employing the WHOQOL-BREF instrument, the research hypothesized that AUD patients would show significantly lower QoL scores across its domains compared to general population norms. The study focused on identifying the QoL domains most impacted by AUD, exploring correlations between QoL scores and AUD background characteristics, and pinpointing intervention areas for patient care improvement. Participants were predominantly males (88.57%) with a middle-aged average of 55.51 years. Educational backgrounds varied, with a notable percentage having attended college (44.29%) or university (17.14%). Regarding marital status, 41.43% were married. Comorbidities were present in 52.86% of the sample, with hypertension being the most common (34.29%). Results showed mean QoL scores in the physical (61.84 ± 16.05), psychological (64.11 ± 17.16), social (60.48 ± 24.85), and environmental (68.44 ± 17.34) domains, revealing a significant diversity in satisfaction levels across these areas. Statistical analyses highlighted marital status as significantly associated with a better QoL in the physical domain, with married, co-habiting, and divorced participants reporting higher scores compared to single ones. In conclusion, while AUD significantly affects the QoL of hospitalized patients in Romania, marital status emerges as a critical factor in mitigating these effects, particularly in the physical domain of QoL. These findings underscore the complexity of AUD’s impact on QoL and the importance of considering sociodemographic factors in patient care practices and interventions. The study contributes valuable insights into the nuanced relationship between AUD and QoL, proposing a foundation for enhancing care outcomes for AUD patients in Romania.

## 1. Introduction

In the context of healthcare, Alcohol Use Disorder (AUD) represents a significant challenge, encompassing a spectrum of issues from mild drinking problems to severe addiction [1,2]. The World Health Organization (WHO) categorizes AUD as a major contributor to the global burden of disease, underlining its impact not just on physical health but also on psychological well-being and social functioning [3,4,5]. In Romania, as in many other countries in Eastern Europe and the Balkan region, the prevalence of AUD poses unique challenges to the healthcare system, necessitating a comprehensive approach to its management [6,7,8]. This is particularly pertinent in hospital settings, where the complexity of AUD can intersect with various health conditions, making the assessment and improvement of quality of life (QoL) in these patients a priority [9,10].

The WHO Quality of Life (WHOQOL) survey emerges as a pivotal tool in this endeavor, offering a multidimensional perspective on the well-being of various population categories [11,12]. It encompasses an array of domains including physical health, psychological state, level of independence, social relationships, personal beliefs, and their relationship to salient features of the environment [13]. Utilizing the WHOQOL survey in hospitalized patients with AUD in Romania allows for a nuanced understanding of the impact of alcohol dependency on various facets of life, facilitating targeted interventions that address both the medical and psychosocial components of the disorder.

Recent studies underscore the importance of integrating QoL assessments into the treatment and management strategies for substance disorders of all types, highlighting a paradigm shift towards patient-centered care [14,15,16]. This approach not only focuses on reducing alcohol consumption and treating comorbid conditions but also emphasizes improving the overall quality of life for these individuals. However, there is a paucity of data regarding the specific QoL outcomes for hospitalized patients with AUD within the Romanian healthcare context, pointing to a gap in the literature and an opportunity for in-depth research in this area.

The relationship between QoL and AUD in Romania (the Balkan region) is further complicated by cultural, economic, and healthcare system factors [17,18]. These elements play a critical role in both the development of AUD and the outcomes of interventions aimed at its treatment. In exploring the challenges of AUD in the Balkan region, it is crucial to consider the nuanced dynamics of alcohol consumption and dependency within these societies. For instance, the prevalence and psychotherapeutic approaches to type III alcoholism in Bulgarian women, as discussed by Ivanova and Giannouli [19], highlight significant gender-specific challenges in the management of AUD. Their findings emphasize the social permissiveness towards drinking in Bulgaria, while also revealing the complex psycho-social factors that contribute to alcohol dependency among women, underscoring the need for targeted psychotherapeutic interventions that consider the distinct cultural and gender dynamics within the region. Thus, understanding the interplay between these factors and QoL in Romanian patients is crucial for developing effective, culturally sensitive approaches to care. Moreover, this understanding can inform policy-making and healthcare service provision, offering insights into how best to support this patient population.

The impact of various substance dependencies, including illicit drugs and prescription medications, universally diminishes QoL across both inpatient and outpatient groups. This broader perspective on addiction underscores the necessity for comprehensive assessment tools that capture the full spectrum of health-related outcomes. To this end, the Short Form Health Survey (SF-36) emerges as a complementary instrument to the WHOQOL-BREF, offering a focused evaluation of health-related QoL that can reveal crucial insights into the physical and mental health dimensions. This approach aligns with other findings that demonstrated that QoL assessments could provide insights into the clinical trajectories of substance use disorders, including the likelihood of maintaining abstinence [20]. Incorporating such tools and perspectives into our study will enrich our understanding and enable more nuanced interventions aimed at enhancing QoL for individuals battling substance dependencies in Romania.

In response to this identified need, the present study aims to assess the quality of life of hospitalized patients with alcohol use disorder in Romania, utilizing the WHOQOL survey. The primary hypothesis of this study is that hospitalized patients with AUD in Romania will exhibit significantly lower QoL scores across all WHOQOL survey domains compared to general population norms. The secondary hypothesis explores the correlation between QoL scores and the background characteristics of patients with AUD, aiming to identify specific domains most impacted by the disorder and identify areas where interventions could be most beneficial. Through this, the study seeks to contribute to the existing body of knowledge and provide a foundation for improving patient care practices and outcomes for individuals with AUD in Romania.

## 2. Materials and Methods

### 2.1. Study Design and Ethical Considerations

This research was structured as a cross-sectional analysis, targeting individuals admitted with AUD at the Psychiatry Clinic of the “Pius Brinzeu” Emergency Clinical Hospital in Timisoara, spanning from 1 January 2023 to 31 December 2023. This study was conducted under the auspices of a collaborative effort involving medical and research faculties with expertise in addiction and mental health, affiliated with the “Victor Babes” University of Medicine and Pharmacy from Timisoara. The investigation was designed to adhere to the highest standards of ethical research conduct, aligning with the principles stipulated in the Declaration of Helsinki—Ethical Principles for Medical Research Involving Human Subjects.

Ensuring compliance with these ethical guidelines, the study was premised on the voluntary participation of patients, with an informed consent process integral to the study’s initiation. This process was designed to thoroughly inform participants about the study’s objectives, the nature of their involvement, the confidentiality of their responses, and their absolute right to withdraw from the study at any point without any adverse implications for their ongoing medical care or personal well-being. The ethical approval for this study was secured from the Ethics Committee of the “Pius Brinzeu” Emergency Clinical Hospital in Timisoara (approval number 2429). 

### 2.2. Inclusion and Exclusion Criteria

The inclusion criteria for this study comprised the following. (1) Diagnosis of alcohol use disorder: individuals must have a formal diagnosis of AUD based on the DSM-5 (Diagnostic and Statistical Manual of Mental Disorders, Fifth Edition) criteria [21], ensuring the study population is homogeneously affected by this condition. (2) Hospitalization status: participants must be currently hospitalized or have been hospitalized for AUD within the study’s designated timeframe, allowing for an accurate assessment of quality of life impacts within a controlled setting. (3) Age: individuals aged 18 years and above are included, reflecting the legal adulthood age in Romania, to ensure participants can provide informed consent. (4) Consent: ability and willingness to provide informed consent for participation in the study, ensuring ethical standards are upheld. (5) Cognitive capacity: participants must possess the cognitive capacity to understand the survey questions and provide meaningful responses, ensuring the reliability of data collected.

Exclusion criteria comprised the following. (1) Severe cognitive impairment: individuals with severe cognitive impairment or neurological conditions that significantly affect comprehension or communication are excluded to ensure the accuracy and reliability of survey responses. (2) Concurrent severe psychiatric disorders: Patients with severe psychiatric disorders other than AUD, which could independently affect their quality of life to a significant extent, are excluded. This ensures that the study’s focus remains on the impact of AUD on quality of life. (3) Critical physical illness: Patients with critical physical illnesses that could independently and significantly affect quality of life measurements are excluded. This criterion is to isolate the effects of AUD from other confounding health conditions. (4) Lack of consent: individuals who are unable or unwilling to provide informed consent are excluded, adhering to the ethical principles governing human research. (5) Language barrier: patients who are unable to understand or communicate in the language(s) in which the WHOQOL survey is administered are excluded, to ensure that participants fully comprehend the survey content and provide valid responses.

### 2.3. WHOQOL

In this study, we utilized the WHOQOL-BREF instrument to evaluate the quality of life in hospitalized patients with AUD in Romania [22,23,24]. The WHOQOL-BREF is a condensed version of the original WHOQOL-100 assessment tool, with a validated translation, including Romanian, which made it a suitable choice for this research.

Our sample size was determined based on a presumed population proportion of 20% of frequent alcohol consumers in the general population, for a margin of error of 10% and a 95% confidence level, resulting in a minimum of 62 participants reaching significance. From the total number of 95 subjects who accepted to participate, 25 provided incomplete questionnaires or decided to quit during enrollment.

The WHOQOL-BREF instrument was administered to all participants in its Romanian-translated version, ensuring cultural appropriateness and comprehension. Participants rated each item on a five-point Likert scale, which ranged from 1 (not at all) to 5 (an extreme amount), facilitating a nuanced capture of their perceptions across the four domains of quality of life. The scoring of the WHOQOL-BREF followed the guidelines provided by the WHO, involving the transformation of scores to a scale of 0–100, which is standard practice for ease of interpretation and comparison. The survey scores were further grouped by domains of physical health, psychological health, social relationships, and environment domains and then calculated by summing the item scores within each domain, followed by a standardization process.

### 2.4. Statistical Analysis

Data management and analysis were conducted utilizing the statistical software SPSS version 26.0 (SPSS Inc., Chicago, IL, USA). A Kolmogorov–Smirnov analysis was performed to determine the normality of data. Continuous variables that were normally distributed were represented as mean ± standard deviation (SD), while categorical variables were expressed in terms of frequencies and percentages. The Student’s *t*-test for comparing two means between the continuous data. The Chi-square test was utilized for the categorical variables. A linear regression analysis was performed to identify the significant factors affecting the QoL in the target population. A *p*-value threshold of less than 0.05 was set for statistical significance. All results were double-checked to ensure accuracy and reliability.

## 3. Results

### Background Characteristics

The study explored the demographic characteristics of 70 hospitalized patients. The average age of the participants was 55.51 years. The majority of participants were in the age range of 41–60 years (62.86%), while the gender distribution showed a significant predominance of men (88.57%). In terms of education, a substantial portion of participants had some form of higher education, with 44.29% attending college and 17.14% having university-level education. Marital status varied among participants: 41.43% were married, making it the most common status. Single individuals comprised 22.86% of the study population, while those in a co-habiting relationship accounted for 11.43%. Comorbidity with alcohol use disorder was noted, with 47.14% of participants not having any comorbidities. However, hypertension was a common comorbidity (34.29%), followed by diabetes mellitus (5.71%) and fatty liver disease (2.86%), as presented in Table 1.

The physical domain score averaged a score of 61.84 (SD = 16.05). The psychological domain showed a slightly higher mean score of 64.11 (SD = 17.16). Social relationships, captured under the social domain, yielded a mean score of 60.48 (SD = 24.85) on the 0–100 scale. The highest mean score was observed in the environmental domain at 68.44 (SD = 17.34) on the 0–100 scale. Lastly, the global score averaged 63.37, as presented in Table 2.

It was observed that age and sex did not present a significant difference in physical domain scores between men and women, with women scoring slightly lower (estimate of −0.55) than men, though this finding was not statistically significant (*p*-value of 0.582). Education level, represented by a per-level increase, showed a minimal and non-significant association with the physical domain scores (estimate of 0.05, *p*-value of 0.856), implying that educational attainment does not substantially influence the perceived physical well-being of the participants in this context. Marital status emerged as significantly associated with the physical dimension of quality of life, as described in Table 3.

Similarly, age did not significantly influence the psychological domain scores, with both the 41–60 years and >60 years age groups showing no statistically significant differences compared to the 18–40 years age group. The estimates of −1.03 and −0.38, respectively, had *p*-values of 0.390 and 0.767. Moreover, sex and education level showed no significant impact on the psychological dimension scores. The comparison between women and men revealed an estimate of −0.23 with a *p*-value of 0.825. Education, assessed as a per level increase, had a minimal and non-significant association with the psychological scores (estimate of 0.12, *p*-value of 0.648).

Marital status, however, was significantly associated with psychological well-being. Participants who were married, in a co-habiting relationship, divorced, or widowed reported higher psychological domain scores compared to single participants, with estimates of 2.57, 3.48, 3.42, and 4.93, respectively. The statistical significance of these findings (*p*-values of 0.002, 0.003, 0.002, and 0.006, respectively) highlights the important role that relationship status plays in the psychological health of individuals hospitalized for alcohol use disorder. The presence of comorbidities, analyzed as per additional condition, did not significantly influence the psychological domain scores (estimate of −0.30, *p*-value of 0.160), as presented in Table 4.

Age showed no significant association with the social dimension scores across different age groups. Similarly, sex and education similarly showed no substantial impact on the social domain scores. Marital status showed potential influence on the social dimension, with married individuals and those in a co-habiting relationship reporting higher scores than single individuals (estimates of 2.53 and 2.26, respectively); however, these associations did not reach statistical significance, as indicated by their *p*-values (0.057 and 0.234, respectively). The presence of comorbidities, analyzed as a per additional condition, had a negligible and non-significant effect on the social domain scores (estimate of 0.05, *p*-value of 0.894), as described in Table 5.

Regarding the WHOQOL-BREF domains, the age of patients with AUD did not significantly impact the environmental domain scores across the different age groups when compared to the younger cohort (18–40 years). Both the 41–60 years and >60 years groups exhibited increases in scores (estimates of 0.81 and 1.35, respectively); however, these differences were not statistically significant (*p*-values of 0.538 and 0.345, respectively). Similarly, gender comparison showed no statistically significant increase in environmental scores for women compared to men (estimate of 0.84, *p*-value of 0.461).

**Figure 1:**
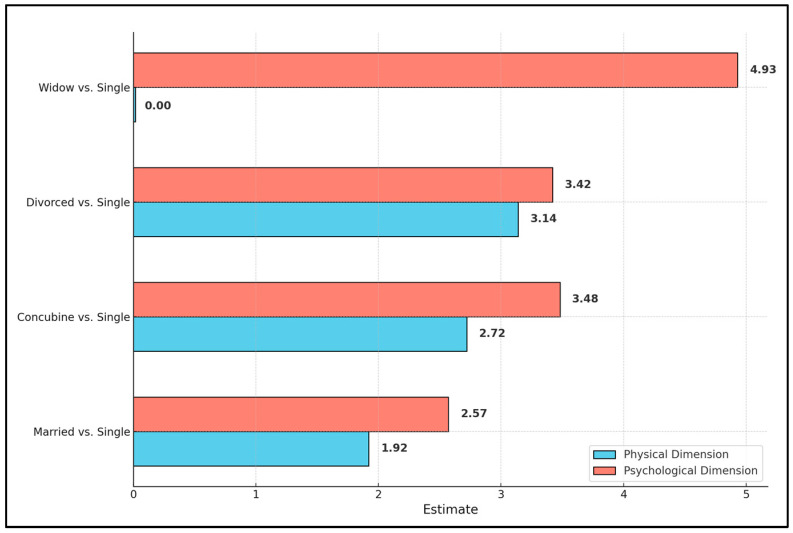
Variables significantly associated with WHOQOL-BREF domains.

Marital status, particularly being married, was the only variable to show a statistically significant association with the environmental dimension scores compared to being single, with an estimate of 1.78 and a *p*-value of 0.045. This finding indicates that married individuals perceive their environment as slightly better than single individuals do, within the context of quality of life. Other relational statuses, such as being in a co-habiting relationship or divorced, showed positive associations, but these did not reach statistical significance. The presence of comorbidities, analyzed per additional condition, showed a negative association with environmental domain scores (estimate of −0.4), with a *p*-value approaching significance (0.094), as seen in Table 6 and Figure 1. 

In a subgroup analysis of hospitalized patients with alcohol use disorder, statistically significant findings indicate that married patients not using any substances exhibit enhanced quality of life across multiple domains compared to single patients. Specifically, married individuals reported significantly higher scores in the physical domain (mean score of 61.56, *p* = 0.018), psychological domain (mean score of 66.32, *p* = 0.002), and environmental domain (mean score of 70.54, *p* = 0.045). These results suggest that marital status, particularly being married, may play a critical role in improving physical and psychological well-being, as well as environmental perceptions, among patients hospitalized for alcohol use disorder (Table 7). 

## 4. Discussion

This study is the first in Romania to examine the quality of life in hospitalized AUD patients, filling a significant gap in regional research. By conducting detailed subgroup analyses on the influence of marital status on quality of life domains, we uncovered notable associations not widely reported elsewhere. These findings are particularly tailored to the unique healthcare landscape of Romania, providing vital insights for local healthcare providers and policymakers to enhance patient care and inform interventions.

In exploring the impact of alcohol use disorder on quality of life among hospitalized patients in Romania, significant findings emerged from the current study, particularly concerning the role of marital status. This variable was significantly associated with enhanced quality of life scores within both physical and psychological domains. Such results underscore the protective effects of relational support systems, hinting at the complex interplay between social relationships and health outcomes in AUD contexts. These insights advocate for the inclusion of relationship-focused interventions in comprehensive AUD treatment frameworks, recognizing the potential of social support in mitigating the disorder’s adverse effects.

Incorporating family and marital therapy into treatment programs for alcohol use disorder has been shown to significantly improve treatment outcomes. Studies have highlighted that engaging family members, especially spouses, in the treatment process increases the likelihood that the individual with AUD will remain in therapy and improves overall recovery outcomes. For instance, Behavioral Couples Therapy (BCT) and Alcohol Behavioral Couple Therapy (ABCT) are notable for their effectiveness [25,26]. These therapies involve partners in the recovery process through joint sessions that enhance communication skills, manage drinking situations, and support changes in drinking behavior. The involvement of partners not only helps reduce alcohol consumption but also enhances relationship functioning and overall family dynamics.

The study’s analysis reveals that, unlike marital status, other demographic factors—age, sex, education, and the presence of comorbidities—do not exhibit a substantial impact on the various dimensions of quality of life. Furthermore, the environmental dimension of quality of life being significantly higher for married individuals reinforces the crucial role of social support. This aspect of quality of life, reflective of individuals’ satisfaction with their living conditions and access to services, points towards the broader environmental and societal factors at play in influencing health outcomes for those with AUD. It implies that improving quality of life in AUD treatment may require strategies that extend beyond the individual to encompass their social and environmental contexts.

Similarly to our findings, Dayal et al.’s study [27] underscored the nuanced impact of psychological factors on the quality of life among individuals with alcohol use disorder, revealing that the attention dimension of impulsivity and anxiety symptoms were significantly associated with the physical health domain of QoL. They found an indirect effect of the attention dimension on physical health as significant, with an effect size of −1.082. Conversely, Colaco et al. [28] provided a broader demographic analysis, demonstrating that domain mean scores for QoL among AUD patients hovered between 50 and 60 on the 0 to 100 scale of the WHOQOL, underscoring the influence of variables such as age, marital status, and years of drinking on these scores.

Other important factors that might interfere with quality of life scores are the socio-economical factors, that were, however, not investigated in the current study. Patkar’s cross-sectional analysis [29] of 100 cases revealed the socio-economic detriments of AUD, highlighting initiation ages between 20–30 and a delayed medical intervention period of 10–12 years, illustrating a chronic trajectory of dependence. This study linked ADS to lower educational attainment, employment levels, and strained personal relationships, underpinning the inverse relationship between alcohol dependence severity and QoL. Conversely, Balhara’s study [30] revealed a high severity of substance use disorder with an average of 8.8 criteria met by individuals. Through WHODAS 2.0, significant impairments were noted in societal participation, household and work-related activities, and cognitive functioning, with negative correlations between disability in social dimensions and QoL measures, particularly psychological health and social relationships. 

Similarly to our study where the majority of patients were men, da Silva Lima et al. [31] focused on male alcohol-dependent patients in Brazil, finding that those with low/moderate dependence reported higher scores across all WHOQoL-BREF domains compared to their severely dependent counterparts, indicating a significant impact of addiction severity on QoL. Their study validated the WHOQoL-BREF’s effectiveness in this demographic, with most correlation coefficients between the WHOQoL-BREF and SF-36 showing significant convergent validity. On the other hand, Iqbal et al. [32] explored HRQoL among warfarin patients in Malaysia, observing higher mean scores in the psychological domain (68.58) than in the physical (61.14), social (63.55), and environmental domains (62.78), with significant associations found between comorbidities and various QoL aspects. Notably, a moderate positive correlation (r = 0.628) between the physical and environmental domains highlighted the interconnected nature of these aspects of patients’ lives.

Other studies also examined the QoL in AUD patients, although focusing on vastly different populations and contexts, as well as different assessment tools. Redwood et al. [33] investigated the relationship between alcohol consumption and health-related quality of life among 1717 Australian adults, finding significant gender differences in alcohol use and its impact on QoL. Males reported higher consumption and a greater risk of alcohol use disorder (AUD) than females, with 20% of males versus 8% of females at greater risk. Particularly, males in regional communities had significantly higher alcohol consumption (AUDIT-C score 6.6) than those in metropolitan areas (AUDIT-C score 4.1), and alcohol consumption was positively associated with QoL in low-moderate risk categories but negative in severe risk categories. In contrast, Baranski et al.’s [34] study on first-year Polish medical students revealed that half of the students experienced distress, with no direct association between alcohol use and general health status. However, females were more likely than males to report higher stress levels, with 68% of the cohort being female. 

In patients with substance use disorder, with and without comorbid psychiatric disorders like schizophrenia and major depressive disorder, studies reveal complex interactions. Hashemzadeh et al. [35] indicated that those with both disorders exhibited more evening-type chronotypes, polydrug use, and notably poorer QOL in domains of physical, psychological, and social health, particularly linked to the severity of schizophrenia and suicide attempts. Conversely, in another study focusing on substance use disorder with and without depression [36], those suffering from both conditions experienced worse sleep quality, including increased sleep latency and daytime dysfunction, and demonstrated poorer QOL, particularly in psychological health-related negatively to the severity of depression. These results underscore the detrimental impact of comorbid psychiatric conditions on circadian rhythms and overall QOL in substance use disorder patients, suggesting that therapeutic strategies should consider both circadian adjustments and mental health management.

These findings contribute to a deeper understanding of the factors influencing quality of life among individuals with AUD, underscoring the significance of social support and marital relationships. The absence of significant associations between quality of life and demographic variables other than marital status suggests that interventions targeting AUD recovery should consider the holistic and relational aspects of patients’ lives. Integrating social support mechanisms into AUD treatment plans could offer a more nuanced and effective approach to enhancing quality of life for individuals affected by this disorder. Although the study results might not be a good representative from a global perspective, they provide a good overview of the population of AUD hospitalized patients from Romania, a country from Eastern Europe that has a homogenous population. Moreover, this is the first study of its kind in our country, focusing on hospitalized patients with AUD which were evaluated on a worldwide-recognized quality of life scale.

Regarding limitations, the cross-sectional design limits the ability to infer causality between AUD and QoL outcomes. The sample size, although adequately fulfilling the calculations and eventually included 95 subjects, faced attrition with 25 patients providing incomplete responses. This attrition may have introduced bias, potentially affecting the study’s representativeness. The reliance on self-reported measures, particularly the WHOQOL-BREF instrument, while validated, is subject to respondents’ interpretation and current psychological state, possibly skewing results towards their perception of QoL rather than objective measures. Another limitation of this study is the reliance on a single scale (WHOQOL) for assessing quality of life in patients with AUD, which may not capture all relevant cognitive and functional aspects influenced by AUD. Additionally, the study’s focus on demographic characteristics without detailed cognitive assessments may overlook significant factors affecting AUD outcomes, as reported by previous studies [37]. While we carefully controlled for several confounding factors, such as age, sex, and significant comorbidities, the potential influence of unmeasured variables, such as socioeconomic status and the duration of hospital stay, cannot be excluded. Additionally, the homogeneous nature of our sample, predominantly middle-aged and male, may limit the generalizability of our findings to other demographics and settings. Finally, as all participants were hospitalized, our results may not reflect the experiences of outpatient or community-dwelling individuals with AUD, suggesting the need for further studies to explore these aspects.

## 5. Conclusions

The study illuminates how AUD profoundly impacts the quality of life of individuals hospitalized in Romania, with a notable observation that marital status plays an essential role in alleviating some of these detrimental effects, especially within the physical aspect of QoL. This discovery highlights the intricate ways in which AUD influences QoL, emphasizing the necessity to integrate sociodemographic considerations into the development of patient care strategies and intervention programs. By delving into the intricate interplay between AUD and QoL, this research offers significant contributions to our understanding, laying a solid groundwork for the improvement of care and support for patients suffering from AUD in Romania.

## Figures and Tables

**Table 1:** Comparison of background characteristics.

Variables	n	%
Age (mean ± SD)	55.51 ± 10.40	-
Age range, n (%)		
18–40 years	7	10
41–60 years	44	62.86
>60 years	19	27.14
Sex, n (%)		
Men	62	88.57
Women	8	11.43
Education, n (%)		
No education	1	1.43
Elementary school	4	5.71
Middle school	14	20.0
College	31	44.29
High school	8	11.43
University	12	17.14
Marital status, n (%)		
Single	16	22.86
Married	29	41.43
Co-habiting	8	11.43
Separated	5	7.14
Divorced	9	12.86
Widow	3	4.29
Comorbidities, n (%)		
No comorbidities	33	47.14
Hypertension	24	34.29
Dyslipidemia	7	10
Fatty liver disease	2	2.86
Diabetes mellitus	4	5.71
Use of other substances		
Tobacco	41	58.57
Recreational drugs	5	7.14

SD—standard deviation.

**Table 2:** Domains and descriptive statistics of the WHOQOL-BREF survey results.

Domains	Domain Scores	
	Score Range 0–100	
	Mean	SD
Physical domain	61.84	16.05
Psychological domain	64.11	17.16
Social domain	60.48	24.85
Environmental domain	68.44	17.34
Global score	63.37	21.59

SD—standard deviation; WHOQOL-BREF—World Health Organization Quality of Life.

**Table 3:** Demographic variables associated with the physical dimension of the WHOQOL-BREF.

Variables	Estimate	SE	t-Value	*p*-Value	95%CI
Age range					
41–60 years vs. 18–40 years	0.79	1.15	0.69	0.495	(−1.51, 3.09)
>60 years vs. 18–40 years	0.78	1.2	0.64	0.522	(−1.63, 3.18)
Sex					
Woman vs. Men	−0.55	0.99	−0.55	0.582	(−2.54, 1.44)
Education					
Per level increase	0.05	0.27	0.18	0.856	(−0.49, 0.59)
Marital status					
Married vs. Single	1.92	0.79	2.44	0.018	(0.35, 3.49)
Comorbidities					
Per additional condition	0.12	0.21	0.54	0.590	(−0.31, 0.54)

CI—confidence interval; SE—standard error.

**Table 4:** Demographic variables associated with psychological dimension of the WHOQOL-BREF.

Variables	Estimate	SE	t-Value	*p*-Value	95%CI
Age range					
41–60 years vs. 18–40 years	−1.03	1.19	−0.87	0.390	(−3.40, 1.35)
>60 years vs. 18–40 years	−0.38	1.29	−0.30	0.767	(−2.96, 2.19)
Sex					
Woman vs. Men	−0.23	1.03	−0.22	0.825	(−2.29, 1.83)
Education					
Per level increase	0.12	0.27	0.46	0.648	(−0.42, 0.66)
Marital status					
Married vs. Single	2.57	0.79	3.25	0.002	(0.99, 4.15)
Comorbidities					
Per additional condition	−0.30	0.21	−1.42	0.160	(−0.73, 0.12)

CI—confidence interval; SE—standard error; WHOQOL-BREF—World Health Organization Quality of Life.

**Table 5:** Demographic variables associated with social dimension of the WHOQOL-BREF.

Variables	Estimate	SE	t-Value	*p*-Value	95%CI
Age range					
41–60 years vs. 18–40 years	−0.03	1.96	−0.01	0.989	(−3.95, 3.89)
>60 years vs. 18–40 years	0.67	2.12	0.32	0.752	(−3.58, 4.93)
Sex					
Woman vs. Men	0.07	1.7	0.04	0.968	(−3.33, 3.47)
Education					
Per level increase	−0.19	0.45	−0.44	0.665	(−1.08, 0.70)
Marital status					
Married vs. Single	2.53	1.3	1.94	0.057	(−0.08, 5.13)
Comorbidities					
Per additional condition	0.05	0.35	0.13	0.894	(−0.66, 0.75)

CI—confidence interval; SE—standard error; WHOQOL-BREF—World Health Organization Quality of Life.

**Table 6:** Demographic variables associated with environmental dimension of the WHOQOL-BREF.

Variables	Estimate	SE	t-Value	*p*-Value	95%CI
Age range					
41–60 years vs. 18–40 years	0.81	1.31	0.62	0.538	(−1.80, 3.42)
>60 years vs. 18–40 years	1.35	1.42	0.95	0.345	(−1.49, 4.18)
Sex					
Woman vs. Men	0.84	1.13	0.74	0.461	(−1.42, 3.10)
Education					
Per level increase	0.23	0.30	0.79	0.433	(−0.36, 0.83)
Marital status					
Married vs. Single	1.78	0.87	2.05	0.045	(0.04, 3.52)
Comorbidities					
Per additional condition	−0.40	0.23	−1.70	0.094	(−0.87, 0.07)

CI—confidence interval; SE—standard error; WHOQOL-BREF—World Health Organization Quality of Life.

**Table 7:** Subgroup analysis of quality of life domains by marital status and substance use in hospitalized patients with alcohol use disorder.

Marital Status	Substance Use	Domain	Mean Score	Standard Deviation	*p*-Value
Single	None	Physical	58.12	16.31	0.653
		Psychological	60.47	17.05	0.721
		Social	57.83	25.00	0.789
		Environmental	65.34	18.64	0.638
	Tobacco	Physical	59.88	15.73	0.565
		Psychological	62.29	16.45	0.473
		Social	59.06	24.12	0.508
		Environmental	67.13	17.85	0.551
	Recreational Drugs	Physical	57.40	16.91	0.740
		Psychological	59.21	17.60	0.762
		Social	56.75	25.33	0.807
		Environmental	64.45	18.97	0.682
Married	None	Physical	61.56	15.08	0.018
		Psychological	66.32	16.23	0.002
		Social	61.10	23.74	0.057
		Environmental	70.54	16.65	0.045
	Tobacco	Physical	62.85	14.52	0.230
		Psychological	67.81	15.95	0.157
		Social	63.29	23.04	0.219
		Environmental	71.27	16.31	0.223
	Recreational Drugs	Physical	60.73	15.43	0.318
		Psychological	65.20	16.74	0.266
		Social	60.08	24.48	0.290
		Environmental	69.13	17.16	0.341

SD—Standard Deviation.

## Data Availability

Data are contained within the article.
